# Defective Speech and Deafness

**Published:** 1895-01

**Authors:** Lawrence M. Stanton

**Affiliations:** New York


### BOOK NOTICES.

Defective Speech and Deafness. By Lillie Eginton Warren. Edgar S. Werner, Publisher, 108 East Sixteenth Street, New York. Price, $1.00.

In this book the author outlines a system of education of the eye, ear, and vocal organs for the deaf which is not only to teach them to speak fluently but to understand readily when spoken to in ordinary language.

In addition to this there are chapters on various forms of speech defects and phases of deafness.

It is not often that a book on any technical subject is read with zest and fresh interest. This little book has the fascination of fiction and yet there is no attempt at popular style. It interests the lay reader as well as the specialist.

One loses himself in the subject of each chapter as in the character of a novel, and having finished the book there is the same feeling of regret, of wishing it continued.

Miss Warren is not only master of the speciality upon which she writes but has the gift of the accomplished teacher—that of imparting knowledge in such a way that its acquisition is pure pleasure and not a necessary bore.

Miss Warren says: "Many an agreeable impression is dispelled when a stranger open his lips and exposes an infantile pronunciation. Clear, crisp articulation is in keeping with the dignity of business, professional, and social life. It is to be expected that childish things have been put away, even if there be lacking the finish and ease in which the cultivated ear delights." The force of this appeals to each one of us. The speaking voice requires more cultivation than it receives, which at present is next to none at all. The educated ear is as keenly alive to the beauty of the voice in speaking as in singing.

Every teacher and parent ought to remember that "correct articulation is a means of mental development, and exercises to insure it have a beneficial effect upon all the nerves, making them more expressive agents of the mind. Correct articulation also leads directly to correct use of language, whereby are revealed culture and refinement."

Ruskin says that the education of the intellect is wasted on some men and for others mischievous, but that the education of the heart is alike good and necessary for all. In line with this truth is Miss Warren's observations, "A child's happy state is absolutely the first essential for securing a warm or affectionate tone of voice, because, even among the hearing, such a condition of mind relieves the vocal bands from unnatural tension. The prompting to action of the organs of speech comes from the affections more than from the intellect. We will to speak before we go through the act of articulating. If moved by pleasurable sensations, the quality of voice is smooth and agreeable, but if any intensity is felt, the tone reveals that condition to the alert listener."

Dumb persons are especially liable to pulmonary troubles on account of undeveloped lungs. "Life is kept in the body by the air received through the nose, and sometimes, unfortunately, through the mouth; but the person who does not know how to exhale the same air in speech or in song, does not fully develop his lungs."

Again the author says: "The deaf child surely has breath enough for vital purposes; why not for vocal?" and upon this question follows the interesting chapter on "Teaching the Dumb to Speak."

Here is a broad view of education with a special emphasis on the auditory senses. One regrets that this is not more generally recognized at the present time. "Remarkable results have followed the careful development of the auditory sense in children who were previously judged to be wholly deaf. Therefore, it is fitting I should urge that hearing receive the special attention it deserves. Instruction is defective which does not include the education of every sense. That which enters by the eye appeals to the intellect or understanding, and thence to the affections; that which enters by the ear appeals directly to the heart as well as to the understanding."

In the chapter on "Dull Pupils," Miss Warren says: "If one has had his development of speech retarded, he is soon in the unfortunate position of having ideas beyond his verbal expression; his vocabulary increases and soon imposes upon his vocal organs the most impossible feats. This is a critical condition, and may, if not properly cared for, lay the foundation for stammering." Thus we are brought to regard the stammerer in a new light, and instead of thinking him somewhat stupid, we see he is often quite the opposite.

The same might be said of many of the "dull pupils in school," who would be found, on examination, to be merely suffering from defective hearing.

Miss Warren describes two unique cases of invented or "pathological" language, and her fine analysis of the cause of defect in these cases is a most valuable contribution to the literature on this subject.

"Their education was at a standstill," she says of these cases. "Efforts had been made to instruct the older boy. The result was utterly discouraging."

These two boys at this time came under Miss Warren's instruction, and she writes: "The boys rapidly acquired the art of arranging words correctly in sentences, and in a few months were able to take their rightful place in school. From extreme unintelligibility they grew into a surprisingly correct and fluent utterance."

The book from beginning to end shows the practical worker, and is full of rich thought. Nothing is overstated; the moderate tone throughout carries conviction. Yet Miss Warren is an enthusiast. The enthusiasm is that born of achieved success, not of misconceived possibilities.

Every specialist, physician, and teacher should read this book, and every intelligent parent, whether or not of deaf children, will find it most useful.

LAWRENCE M. STANTON, M. D.,
New York.

